# Supplementary material for: Challenges and solutions in determining urolithiasis caseloads using the digital infrastructure of a clinical data warehouse
Source: PLoS One. 2026 Jan 23;21(1):e0341068. doi: 10.1371/journal.pone.0341068 (PMC12829838; doi:10.1371/journal.pone.0341068)
Supplement: S1 Appendix — (PDF) [file pone.0341068.s001.pdf]

S1 Appendix. DRG merging rules and diagnosis reclassification

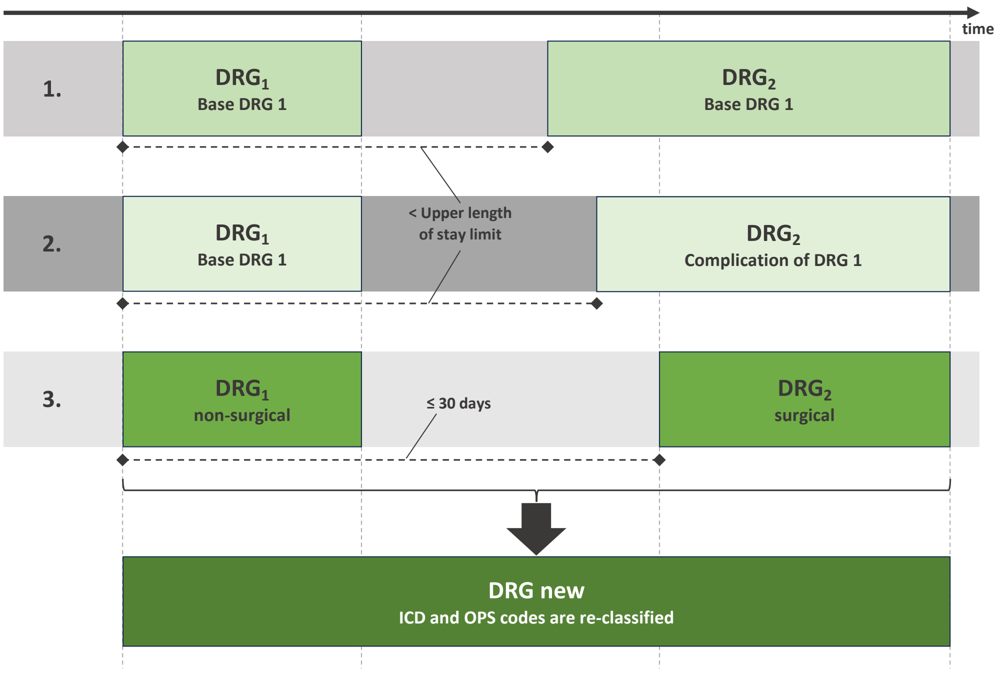

According to DRG guidelines, cases will be merged in three situations: (1) re-admission within the upper length of stay limit when cases are coded with same base-DRG code or (2) the second case is coded with a complication of the first case, and (3) re-admission within 30 days and same DRG-base-code, assuming the first case was non-surgery and the second with surgery. There are special DRG codes, where cases are not merged (e.g. dialysis or radiation therapy without surgery). [1] The upper length of stay limit (in German "obere Grenzverweildauer") is the maximum number of inpatient days covered by a standard DRG payment. [2] In our use case urolithiasis, for example, a first case with ureteral stent and a second case with a surgical removal of a urinary stone 14 days later would be merged into one case. Both cases have the same DRG-base-code, the first case was non-surgical and the second surgical. When these two cases are combined, the data are re-evaluated and are given one new DRG code for the merged cases. During this process, all diagnoses and procedures from both stays are considered jointly. The ICD-10 codes are rearranged so that the clinically dominant condition is defined as the main diagnosis, while the others are retained as secondary diagnoses. When a secondary urolithiasis diagnosis is clinically more relevant than the primary diagnosis, a reclassification may lead to differences between the original individual cases and the merged case.

References

1. Institut für das Entgeltsystem im Krankenhaus (InEK). Fallpauschalenkatalog 2025; 2025.

2. GKV-Spitzenverband and Verband der Privaten Krankenversicherung and Deutschen Krankenhausgesellschaft. Vereinbarung zum Fallpauschalensystem für Krankenhäuser für das Jahr 2024 (Fallpauschalenvereinbarung 2024 – FPV 2024) vom 06.11.2023; 2024. Available from: https://www.dkgev.de/fileadmin/default/Mediapool/2_Themen/2.2_Finanzierung_und_Leistungskataloge/2.2.1._Stationaere_Verguetung/2.2.1.4._Abrechnungsbestimmungen/FPV_2025.pdf.
