# Supplementary material for: Challenges and solutions in determining urolithiasis caseloads using the digital infrastructure of a clinical data warehouse
Source: PLoS One. 2026 Jan 23;21(1):e0341068. doi: 10.1371/journal.pone.0341068 (PMC12829838; doi:10.1371/journal.pone.0341068)
Supplement: S1 Fig — (PDF) [file pone.0341068.s002.pdf]

S1 Figure. Overview of query logic for algorithmic case extraction

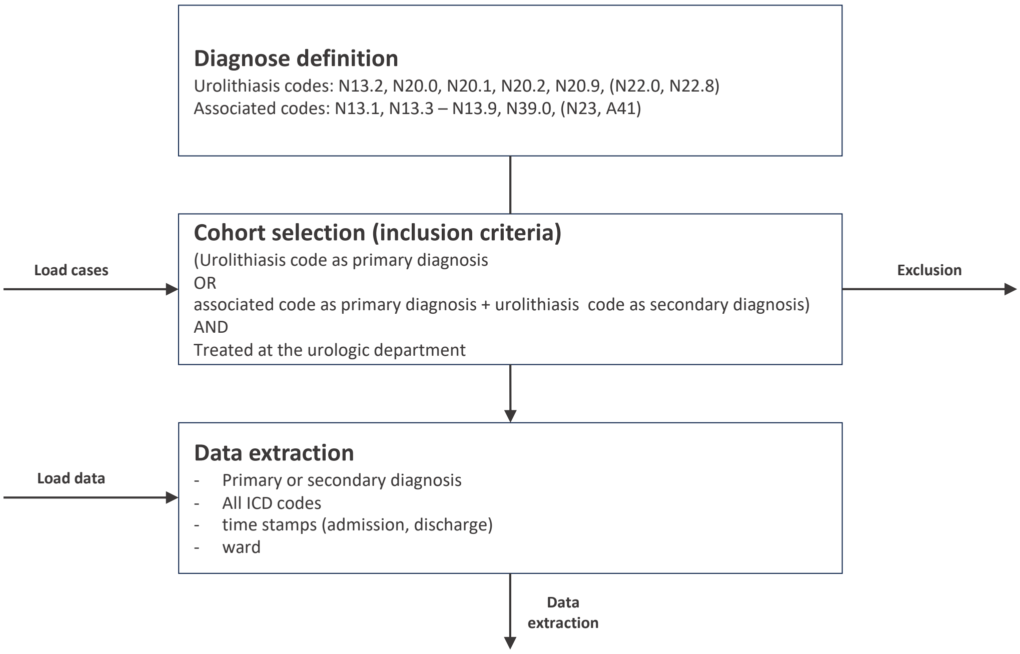

The script for algorithmic extraction (Mode 2 and 3) is structured in three parts: defining urolithiasis and associated codes, selecting cases according to the case definition, and extracting relevant data of cases (ICD-10 codes, time stamps, information about the ward). Mode 3 did not include information about wards.
