# Supplementary material for: Challenges and solutions in determining urolithiasis caseloads using the digital infrastructure of a clinical data warehouse
Source: PLoS One. 2026 Jan 23;21(1):e0341068. doi: 10.1371/journal.pone.0341068 (PMC12829838; doi:10.1371/journal.pone.0341068)
Supplement: S1 Table — (PDF) [file pone.0341068.s003.pdf]

S1 Table. Clinical characteristics of merged cases

According to the DRG guidelines, the main risk factor for case merging is a case distance (the number of days between a first case's discharge date and a second case's admission date) of under 30 days. Our sub-sample consists of five patients: four patients were missing one case, and one patient appeared as an additional case in the reimbursement data. All case distances of the sub-sample fall below this 30-day threshold.

**Characteristics**	**Count**
**Merged cases**	
Patients (N)	5
Cases (n)	9
**Case definition**	
Primary	9 (100%)
Secondary[1]	3 (33.3%)
**Complications, ICD-10**	
N39.0	3 (33.3%)
R31	1 (11.1%)
**Other diagnoses (with $n > 5$)**	
Z11	7 (77.8%)
Z96.0	4 (44.4%)
**Treatment, OPS code**	
8-137[2]	8 (88.9%)
**Timing**	
Case length in days (mean, range)	2.67 [1 - 4]
Case distance in days (mean, range)	9.2 [2 - 15]

[1] Fullfilling primary and secondary case criteria.
[2] We observed also other OPS codes (less than 5 cases)
