# Supplementary material for: Challenges and solutions in determining urolithiasis caseloads using the digital infrastructure of a clinical data warehouse
Source: PLoS One. 2026 Jan 23;21(1):e0341068. doi: 10.1371/journal.pone.0341068 (PMC12829838; doi:10.1371/journal.pone.0341068)
Supplement: S1 Checklist — (PDF) [file pone.0341068.s004.pdf]

S1 Checklist. Ensure suitability for case extraction from performance data

- **Include essential patient encounter information:** admission date, discharge date, and ward information, as well as diagnoses provided in the form of ICD codes.
- **Use complete ICD-10 codes:** ensure all ICD-10 codes are provided at the full four-digit level and clearly categorized into admission vs. discharge as well as primary vs. secondary diagnoses.
- **Verify completeness of ward coverage:** confirm that all relevant wards or clinics involved are represented in the dataset.
