# Supplementary material for: Challenges and solutions in determining urolithiasis caseloads using the digital infrastructure of a clinical data warehouse
Source: PLoS One. 2026 Jan 23;21(1):e0341068. doi: 10.1371/journal.pone.0341068 (PMC12829838; doi:10.1371/journal.pone.0341068)
Supplement: S2 Checklist — (PDF) [file pone.0341068.s005.pdf]

S2 Checklist. Rules for manual extraction

1. Identify all relevant wards and include them in the extraction.
2. Ensure accurate date information on hospital admission and discharge (length of stay > 0 hour/min/day).
3. Check primary diagnosis before secondary diagnoses, which are often numerous.
4. Define data formats before and be consistent (e.g. N20 vs. N.20).
5. Limit data collection to relevant variables.
